# Anti-inflammatory and Antinociceptive Activity of Ouabain in Mice

**DOI:** 10.1155/2011/912925

**Published:** 2011-05-26

**Authors:** Danielle Ingrid Bezerra de Vasconcelos, Jacqueline Alves Leite, Luciana Teles Carneiro, Márcia Regina Piuvezam, Maria Raquel Vitorino de Lima, Liana Clébia Lima de Morais, Vivian Mary Rumjanek, Sandra Rodrigues-Mascarenhas

**Affiliations:** ^1^Laboratório de Tecnologia Farmacêutica, Departamento de Fisiologia e Patologia, Centro de Ciências da Saúde, Universidade Federal da Paraíba, João Pessoa 58059-900, Brazil; ^2^Departamento de Biologia Molecular, Centro de Ciências Exatas e da Natureza, Universidade Federal da Paraíba, João Pessoa 58059-900, Brazil; ^3^Instituto de Bioquímica Médica, Centro de Ciências da Saúde, Universidade Federal do Rio de Janeiro, Rio de Janeiro 21941-902, Brazil

## Abstract

Ouabain, an inhibitor of the Na^+^/K^+^-ATPase pump, was identified as an endogenous substance of human plasma. Ouabain has been studied for its ability to interfere with various regulatory mechanisms. Despite the studies portraying the ability of ouabain to modulate the immune response, little is known about the effect of this substance on the inflammatory process. The aim of this work was to study the effects triggered by ouabain on inflammation and nociceptive models. Ouabain produced a reduction in the mouse paw edema induced by carrageenan, compound 48/80 and zymosan. This anti-inflammatory potential might be related to the inhibition of prostaglandin E2, bradykinin, and mast-cell degranulation but not to histamine. Ouabain also modulated the inflammation induced by concanavalin A by inhibiting cell migration. Besides that, ouabain presented antinociceptive activity. Taken these data together, this work demonstrated, for the first time, that ouabain presented *in vivo* analgesic and anti-inflammatory effects.

## 1. Introduction

Ouabain, one of the oldest drugs used for treatment of cardiac insufficiency, is classically known as a specific inhibitor of the plasma membrane Na^+^/K^+^-ATPase [1]. Originally isolated from plants, it was demonstrated that mammals naturally produce an endogenous analogue of ouabain, a hormone synthesized in the adrenal glands, hypothalamus, and pituitary and found circulating in the plasma [2–5]. Accumulating evidence suggests that circulating levels of endogenous ouabain are modulated by stress conditions. Elevated plasma levels of ouabain were found in hypertensive patients and after physical exercise in different species [6–8]. 

The steroid ouabain is capable of modulating many aspects of the immune system, being considered as an immunomodulatory molecule [9]. Ouabain, *in vitro*, inhibits mitogen-induced thymocyte and lymphocyte proliferation [10, 11], inhibits the generation of lymphokine-activated killer (LAK) activity induced by IL-2 [12], increases intracellular calcium levels [13], increases CD69 expression [14] and acts both *in vitro* [15] and *in vivo* [16] synergistically with corticoids. *In vitro*, ouabain increases glucocorticoid-induced plasma membrane depolarization in lymphoid cells [9]. Besides that, ouabain downregulates mCD14 expression on monocytes, which is related to inflammatory response against several pathogens [17] and induces IL-1 *β* production on human mononuclear cells [18].

Additionally, this molecule is also capable of inducing the activation of various signal transduction cascades that are independent of changes in intracellular Na^+^ and K^+^ concentrations, involving the Ras/Raf/mitogen-activated protein kinase (MAPK) cascade, transactivation of epidermal growth factor receptor (EGFR), and protein kinase C [17, 19–23]. In murine thymocytes, ouabain decreased the levels of phosphorylated MAPK p38 and nuclear factor of activated T-cells (NFATc1) induced by the mitogen concanavalin A [24].

Besides the effects described in the immune system, little is known about the role of ouabain in inflammatory processes. During inflammation, a complex program of intracellular signal transduction and transcription events, driven by multiple proinflammatory mediators and cytokines, is activated. The acute inflammation is characterized by exudation of protein-rich fluid, edema, vasodilatation, and cell migration, primarily neutrophils, into the site of injury [25]. It was reported that ouabain suppressed the production of the pro-inflammatory cytokines IL-6 and TNF-*α* stimulated with LPS both *in vitro *and *in vivo* [26]. In addition, cardiac glycoside drugs inhibits TNF-*α*/NF-*κ*B signaling pathway, which is a central common regulator for the process of inflammation [27].

The aim of the present study was to investigate the role of ouabain in acute peripheral inflammation induced by intraplantar and intraperitoneal injection of different phlogistic agents and in algesic processes.

## 2. Material and Methods

### 2.1. Animals

Female swiss albino mice (2 months old) were housed in a temperature-controlled room and received water and food *ad libitum*. After manipulation, euthanasia was employed by cervical dislocation. The study was performed according to the Guidelines on Ethical Standards for Investigation of Experimental Pain in Animals [28], after approval of protocol no. 0407/08 by the Institutional Ethics Committee of Laboratório de Tecnologia Farmacêutica.

### 2.2. Treatment with Ouabain

In all experiments, 0.56 mg/kg ouabain [16] or phosphate buffered saline (PBS) was given intraperitoneally (i.p.) for three consecutive days. A dose-response curve was also performed using 0.10 mg/kg and 0.31 mg/kg ouabain, and 0.56 mg/kg ouabain was given i.p. for one and two consecutive days.

### 2.3. Inflammatory Paw Edema

To induce inflammation, mice received subcutaneous injections in the plantar surface [29] of carrageenan (2.5%), compound 48/80 (2 *μ*g/paw), zymosan (1%), histamine (100 *μ*g/paw), prostaglandin E2 (5 *μ*g/paw), and bradykinin (6 nmol/paw) in 20 *μ*L of phosphate buffered saline (PBS) in the right (ipsilateral) hindpaw and 20 *μ*L of PBS in the left (contralateral) hindpaw. Captopril (5 mg/kg) was used 1 h prior to bradykinin challenge in order to prevent the action of kininases [30]. Ouabain last treatment was injected i.p. one hour before intraplantar injections of phlogistic agents. All the reagents were obtained from Sigma. Hindpaw edema was measured with a digital micrometer (Instrutemp 070393611) [31] and is expressed as the difference of thickness (mm) between the stimulated and the saline-injected paw. 

Dexamethasone (0.5 mg/kg), indomethacin (10 mg/Kg) and salbutamol (10 mg/kg) were used as antiinflammatory controls and injected i.p. or subcutaneous (s.c.) one hour before intraplantar injections.

### 2.4. Peritoneal Inflammation Model

Mice were injected intraperitoneally with 60 ug Concanavalin A diluted in sterile saline solution to a final injection volume of 0.1 mL one hour after the last ouabain administration. After 24 h, animals were sacrificed and peritoneal lavage was performed with 10 mL of sterile PBS [32–34]. The fluid was removed for total and differential cell counts and results were reported as cell number/mL of peritoneal wash. Differentiation of leukocyte subpopulations in peritoneal lavage fluids was performed on cytospin preparations stained with Wright-Giemsa solution (Sigma, St. Louis, Mo, USA). For each slide a minimum of 100 cells was counted microscopically under 1000 magnification.

### 2.5. Acetic Acid Induced Writhing Test

Acetic acid administration causes irritation resulting in painful contortions followed by extension of hind limbs [35]. The animals were injected i.p. with 0.1 mL/10 g of 0.8% (v/v solution) acetic acid one hour after the last treatment with ouabain (0.56 mg/kg). Morphine (6 mg/Kg) (i.p.) was used for analgesia. Ten minutes after the administration of acetic acid, mice were placed in separate boxes and the number of abdominal writhes was counted for 10 minutes. The antinociceptive activity was expressed as the reduction in the number of abdominal writhes when compared to control animals.

### 2.6. Hot Plate

Animals were placed on a hot plate maintained at 52 ± 1°C. The time elapsed between placing the animal on the hot plate, and the animal either licking its fore or hind paws or jumping on the surface was considered the response latency [36]. Mice with baseline latencies of more than 15 s were excluded from the study. Response latency testing was measured 30, 60 and 120 minutes after the last treatment with Ouabain (0.56 mg/kg), saline or the positive control, morphine (10 mg/Kg). The cutoff time for the hot-plate test latency was set at 30 s to avoid tissue injury. All animals were brought to the test room at least 1 h prior to the experiments and were not tested more than once.

### 2.7. Possible Involvement of Opioid Receptors

In order to investigate the involvement of the opioidergic system in ouabain-induced antinociception, separate groups of mice were pretreated with nonselective opioid receptor antagonist, naloxone (5 mg/kg, s.c.), which was injected 15 min before i.p. administration of ouabain (0.56 mg/kg) and morphine (10 mg/kg, i.p.), and tested using the hot plate test [37].

### 2.8. Elevated Plus maze

The apparatus is comprised of two open arms (35 × 5 cm) and two closed arms (30 × 5 × 15 cm) that extended from a common central platform (5 × 5 cm). The entire maze was elevated to a height of 30 cm above floor level [38]. One hour after ouabain administration, mice were placed in the central region of the plus-maze apparatus. Diazepan (0.5 mg/Kg) was administred 30 min before the test and used as positive control.

### 2.9. Statistical Analysis

All data were expressed as mean ± S.E.M. and analyzed by software Graphpad Prism using Student's *t*-test followed by unpaired test or ANOVA followed *Dunnett's *or *Mann Whitney test*, and the results were considered significant if *P* < .05.

## 3. Results

### 3.1. Effect of Ouabain on Carrageenan, Zymosan, and 48/80-Induced Mice Paw Edema

**Figure 1:**
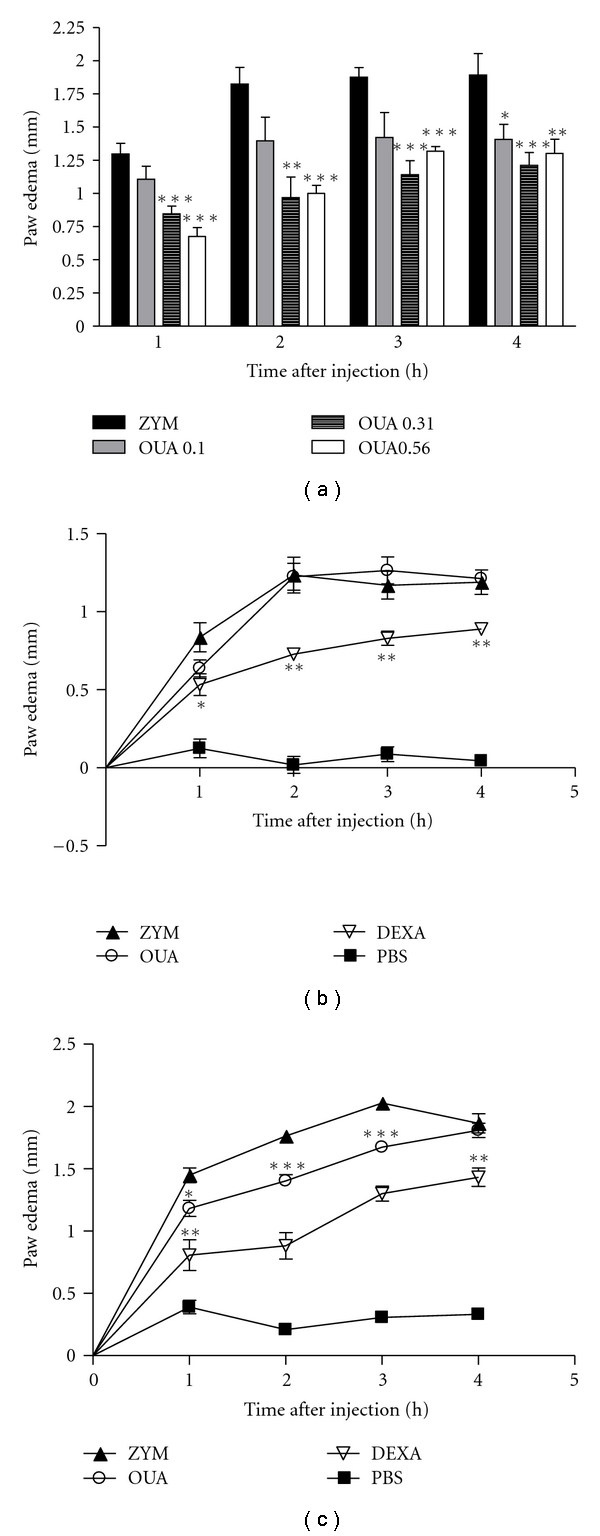
Ouabain dose-response curve and administration for 1 and 2 consecutive days. Mice were pretreated with 0.10 mg/kg, 0.31 mg/kg and 0.56 mg/kg ouabain for 3 consecutive days (a) or with 0.56 mg/kg ouabain for 1 (b) and 2 consecutive days (c); one hour after the last ouabain treatment, mice received intraplantar injections of zymosan in 20 *μ*L phosphate buffered saline (PBS) in the right hindpaw and 20 *μ*L of PBS in the left hindpaw. Each point represents the mean of eight animals. Dexametasone (DEXA, 0.5 mg/kg) was used as antiinflammatory control and injected i.p. one hour before intraplantar challenge. Asterisks denote the significance levels compared with ZYM group. Data were expressed as mean ± S.E.M. and analyzed by software Graphpad Prism using Student's *t*-test followed by unpaired test. *P < .05, **P < .01, and ***P < .001.

**Figure 2:**
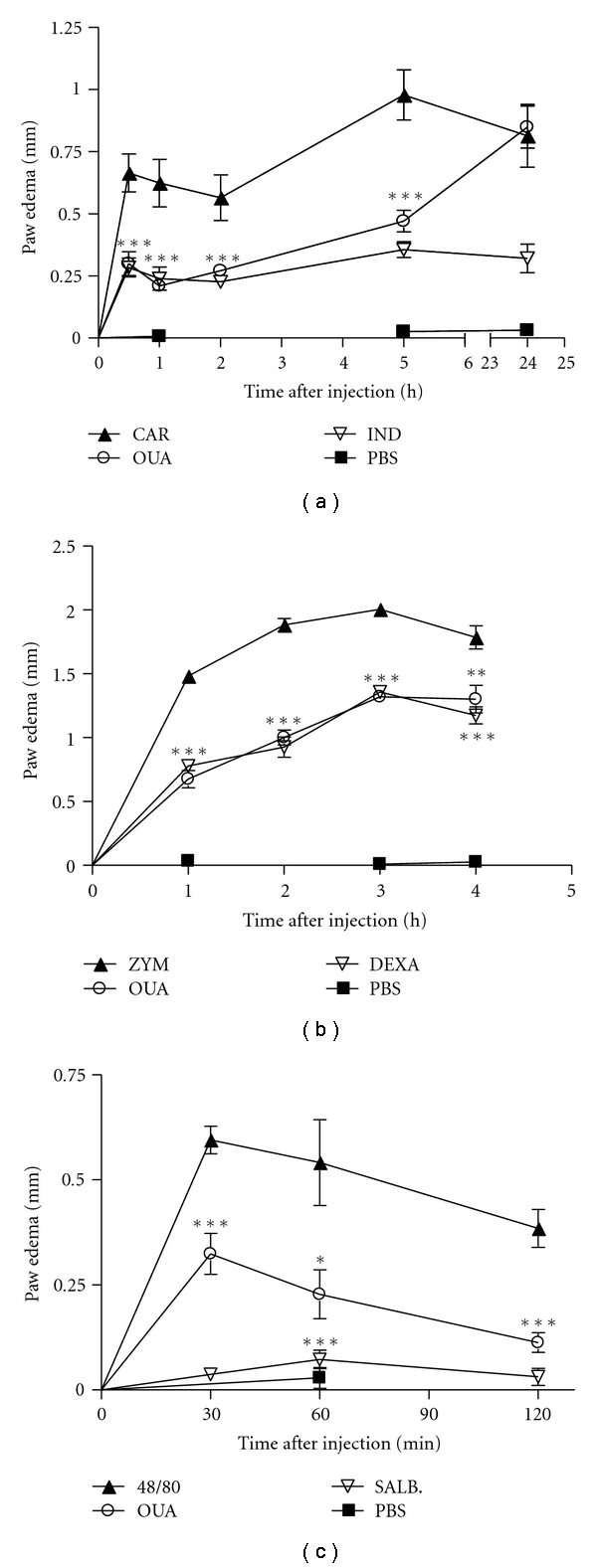
Effect of the pretreatment with Ouabain (OUA, 0.56 mg/kg) administered intraperitoneally on paw edema triggered by carragenan (CAR), zymosan (ZYM), and compound 48/80 in mice. Mice received intraplantar injections of carrageenan (2.5%, (a)), zymosan (1%, (b)), or compound 48/80 (2 *μ*g/paw, (c)) in 20 *μ*L phosphate buffered saline (PBS) in the right hindpaw and 20 *μ*L of PBS in the left hindpaw. Each point represents the mean of eight animals. Indomethacin (IND, 10 mg/Kg), dexametasone (DEXA, 0.5 mg/Kg), and salbutamol (SALB, 10 mg/kg) were used as antiinflammatory controls and injected i.p. one hour before intraplantar challenge. Asterisks denote the significance levels compared with CAR, ZYM, or compound 48/80 group. Data were expressed as mean ± S.E.M. and analyzed by software Graphpad Prism using Student's *t*-test followed by unpaired test *P < .05, **P < .01, and ***P < .001.

The paw edema induced by carrageenan and zymosan involves various mediators such as histamine, bradykinin, and prostaglandins [39, 40]. Although 0.10 mg/kg ouabain was without effect, 0.31 mg/kg and 0.56 mg/kg ouabain prevented zymosan edema formation (Figure 1(a)). On the other hand, ouabain was not able to inhibit zymosan-induced paw inflammation when administered only one day prior experiment (Figure 1(b)). Furthermore, when ouabain was given for two days prior experiment, it did not interfere in the edema present 4 h after zymosan (Figure 1(c)). Ouabain 0.56 mg/kg injected for three consecutive days, prevented zymosan edema formation at the 1st (54.4%), 2nd (47.1%), 3rd (34.7%), and 4th h (26.9%) after treatment, as well as did 0.5 mg/kg dexamethasone (Figure 2(b)). Similarly to what was observed with zymosan, carrageenan-induced paw edema was also significantly reduced in a time-dependent manner by the treatment of 0.56 mg/Kg ouabain at 30 min (54.9%), 1st (66.4%), 2nd (51.0%), and 5 h (51.9%) after carrageenan treatment (Figure 2(a)). On the other hand, ouabain did not interfere in the edema present 24 h after carrageenan injection. A significant antiinflammatory effect of indomethacin at the dose of 10 mg/Kg was observed at all times studied. Paw edema induced by compound 48/80 was short lasted, with a peak 30 min after injection. Ouabain significantly inhibited the edema, but to a lesser extent than salbutamol, at 30 (69.1%), 60 (79.7%), and 120 min (49.7%) after compound 48/80 injection (Figure 2(c)).

### 3.2. Effect of Ouabain on Paw Edema Triggered by Different Inflammatory Mediators

**Figure 3:**
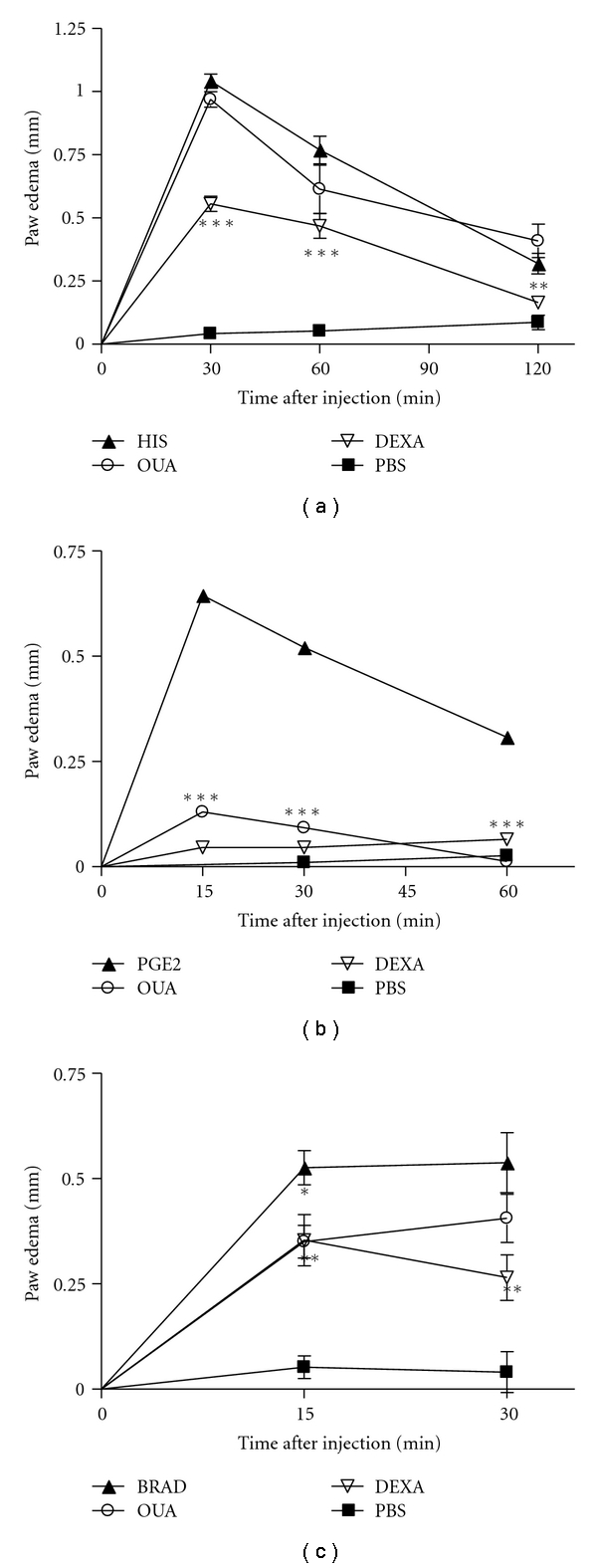
Effect of the pretreatment with Ouabain (OUA, 0.56 mg/kg) administered intraperitoneally on paw edema triggered by histamine (HIS), prostaglandin E2 (PGE2) and bradykinin (BRAD). Mice received intraplantar injections of histamine (100 *μ*g/paw, (a)), prostaglandin E2 (5 *μ*g/paw, (b)) or bradykinin (6 nmol/paw, (c)) in 20 *μ*L of phosphate buffered saline (PBS) in the right hindpaw and 20 *μ*L of PBS in the left hindpaw. Each point represents the mean of eight animals. Dexametasone (0.5 mg/kg) was used as antiinflammatory control and injected i.p. one hour before intraplantar challenge. Data were expressed as mean ± S.E.M. and analyzed by software Graphpad Prism using Student's *t*-test followed by unpaired test *P < .05, **P < .01, and ***P < .001.

The results in Figure 3(a) indicate that ouabain was incapable to inhibit the edema induced by histamine at any of the times studied. On the other hand, the administration of ouabain produced a significant reduction in the mouse paw edema induced by prostaglandin E2 and bradykinin (Figures 3(b) and 3(c)). A significant antiedema effect of ouabain was observed at 15 (79.8%), 30 (82.1%) and 60 min (96.0%) after prostaglandin E2 challenge and at 15 (34.0%) but not at 30 min after bradykinin challenge.

### 3.3. Effect of Ouabain Treatment on the Cellular Influx into the Peritoneal Cavity

**Figure 4:**
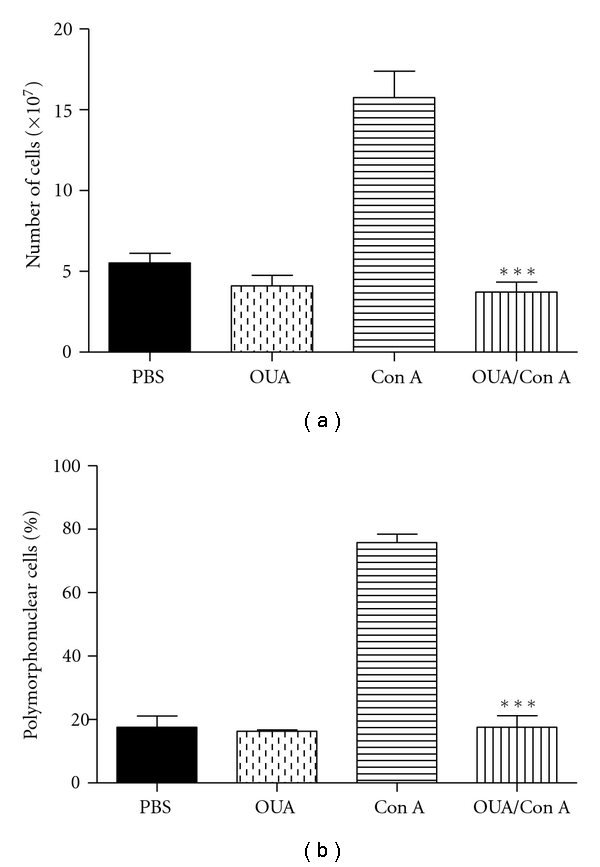
Effect of the pretreatment with Ouabain (OUA, 0.56 mg/kg) administered intraperitoneally on peritoneal inflammation triggered by Concanavalin A (ConA, 60 *μ*g/300 *μ*L) on total (a) and differential cell counts (b). Each bar represents the mean of nine animals. Asterisks denote the significance levels compared with Con-A values. Data were expressed as mean ± S.E.M. and analyzed by software Graphpad Prism using Student's *t*-test followed by unpaired test **P < .01 and ***P < .001.

Peritoneal inflammation was induced and the number of cells recruited into the peritoneal cavity was measured as an indication of the degree of inflammation. As shown in Figure 4(a), the administration of ouabain alone had no effect on the number of resident peritoneal cells in unchallenged mice. However, after induction of inflammation, treatment with ouabain led to a 70%–80% reduction in the total cell numbers in the peritoneal cavity after 24 h, as a reflex of the inhibition of polymorphonuclear leukocytes (Figure 4(b)). These data are consistent with an attenuated inflammatory response observed.

### 3.4. Effect of Ouabain on Acetic Acid-Induced Writhing Response

**Figure 5:**
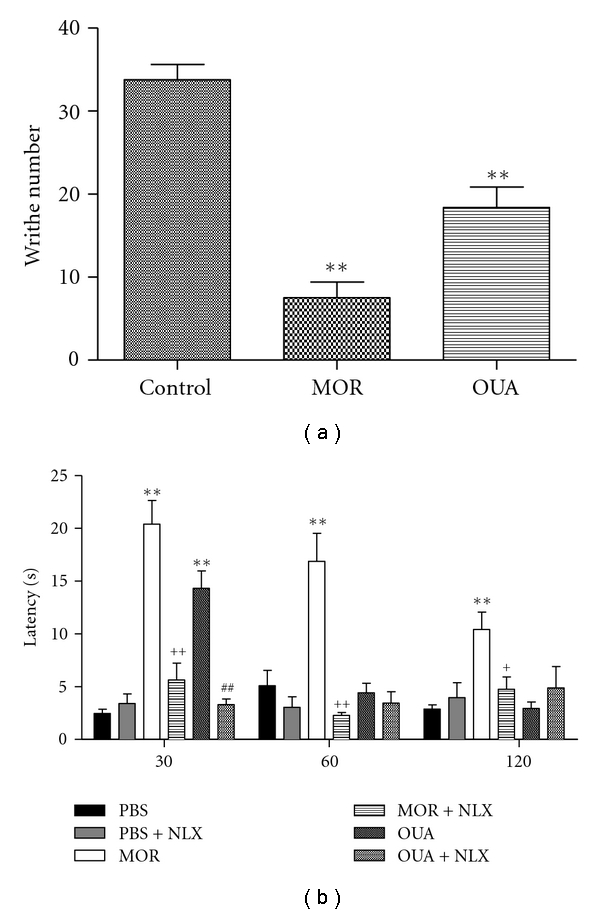
Effect of the pretreatment with Ouabain (OUA, 0.56 mg/kg) administered intraperitoneally on acetic acid-induced writhing and hot plate test in mice. Each bar represents the mean of six or ten animals. Morphine (MOR, 10 mg/kg) administered intraperitoneally was used for analgesia. Naloxone (NLX, 5 mg/kg), an opioid antagonist, was used to test the involvement of opioid receptors. Asterisks denote the significance level compared with control values; # compared with the Ouabain group and + compared with the morphine group. Data were expressed as mean ± S.E.M. and analyzed by software Graphpad Prism using (ANOVA) followed *Dunnett's test*. *P < .05; **P < .01, and ***P < .001.

The results of acetic acid-induced writhing responses in mice, which indicate ouabain's analgesic activity, were presented in Figure 5(a). It was demonstrated that ouabain caused a significant inhibition (45%) on the writhing responses induced by acetic acid when compared to the control group, as well as did the analgesic drug, 6 mg/kg morphine (78%).

### 3.5. Effect of Ouabain on the Hot Plate Test and Involvement of Opioid Receptors

The results of the hot plate test revealed that the latency time was increased (74%) when animals were tested 30 min after the last injection of ouabain. Pretreatment of mice with naloxone inhibited the antinociceptive effect triggered by ouabain (around 77%). On the other hand, ouabain was not capable to interfere in the latency observed after 60 or 120 min. The analgesic drug, morphine (10 mg/kg) modulated the pain, increased the latency time (more than 70%), and it was significantly reversed by naloxone (70%), (Figure 5(b)). 

### 3.6. Effect of Ouabain on Elevated Plus-Maze Test

**Figure 6:**
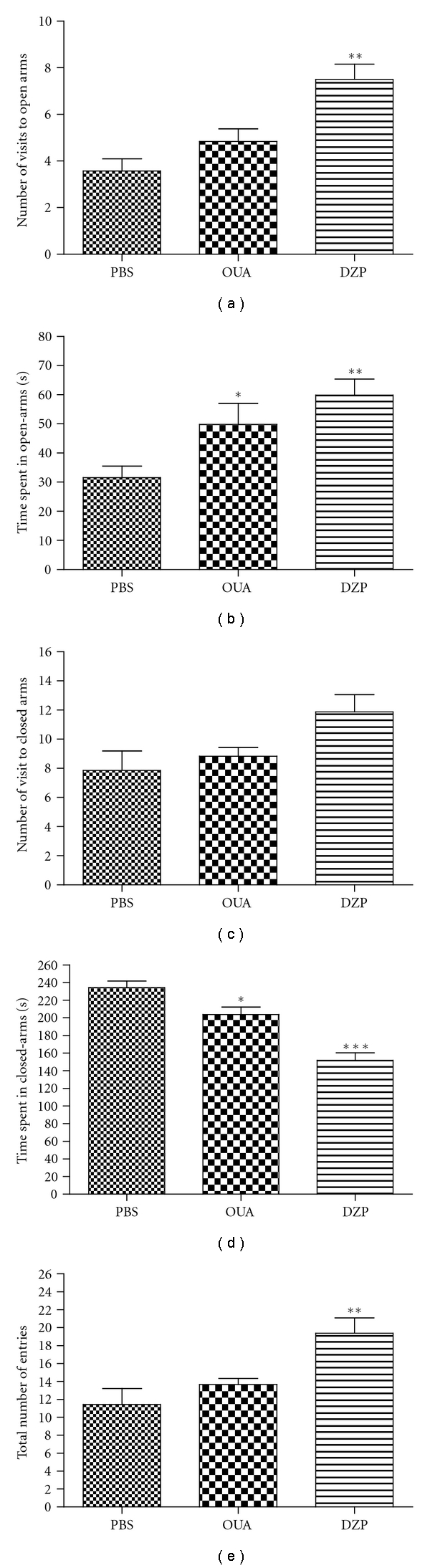
Effect of the pretreatment with Ouabain (OUA 0.56 mg/kg) administered intraperitoneally on elevated plus-maze test in mice. (a) Number of visit to open-arms; (b) Time spent in open-arms; (c) Number of visit to closed-arms; (d) Time spent in closed arms; (e) Total numbers entries. Each bar represents the mean of seven or eight animals. Diazepan (DZP 0.5 mg/Kg) was used as positive control. Data were expressed as mean ± S.E.M. and analyzed by software Graphpad Prism using ANOVA followed Mann Whitney test or Student's *t*-test followed by unpaired test: *P < .05, **P < .01, and ***P < .001.

The results of the elevated plus maze test revealed that ouabain did not interfere in the numbers of entries in open arms but increased the time spent (Figures 6(a) and 6(b)). Additionally, the number of visits was not modified, and the time spent in closed arms was decreased by ouabain (Figures 6(c) and 6(d)). Ouabain did not interfere in total number of entries (Figure 6(e)). The positive control (0.5 mg/Kg diazepan), increased the number total of entries as well the number of entries and the time spent in open arms and decreased the time spent in closed arms.

## 4. Discussion

In agreement with the immunosuppressive effects that have been previously observed, in the present work, ouabain inhibited inflammation and nociception. To study the role of ouabain in the inflammatory process, initially we used the carrageenan and zymosan-induced paw edema. These models induce inflammatory responses, including edema formation, neutrophil infiltration, and the development of hyperalgesia [41]. 

Ouabain administered for three consecutive days was able to inhibit zymosan edema formation at all periods studied. On the other hand, ouabain was not able to inhibit zymosan-induced paw inflammation when administered only one day prior experiment. Furthermore, when ouabain was given for two days prior experiment, it did not interfere in the edema present 4 h after zymosan. Despite the fact that ouabain was effective at the 0.31 mg/kg concentration, the dose of 0.56 mg/kg was chosen because, using a different model, in a previous *in vivo* work, this concentration was effective [16]. The intraplantar administration of zymosan activates of the complement system [42] promotes increased expression of COX-2 and prostaglandin production, mainly of type E2, and increases the production of nitric oxide [39]. A controversy exists regarding the role played by ouabain on mast cell degranulation. Stimulatory and inhibitory effects were reported [43, 44]. Some data have also demonstrated that ouabain had no effect on mast cell degranulation [45]. In this work, the edema induced by mast cell degranulation using compound 48/80 was also inhibited. 

In agreement with the results using zymosan, ouabain administered for three consecutive days was also able to inhibit carrageenan induced edema formation. Henriques and coworkers [46] showed that carrageenan injection into the mouse paw induces a biphasic edema that develops in the first 6 h, followed by a second phase, which starts at 24 h. The early phase is mainly mediated by histamine, serotonin, and by an increasing synthesis of prostaglandins such as prostaglandin E2. Recent studies have shown that carrageenan also induces peripheral release of nitric oxide (NO) sustained by TNF-*α*, IFN-y, and IL-1. These cytokines have been shown to induce iNOS in a variety of cells [47]. The late phase is mainly sustained by prostaglandin release and NO [48]. In the present work, we found that ouabain inhibits the inflammation after 30 min, 1, 2, and 6 h but not after 24 h of carrageenan challenge. The antiinflammatory effect observed in the first phases could be related to the suppression of the pro-inflammatory cytokines IL-6 and TNF-*α* [26] and by the blockade of the TNF-*α*/NF-*κ*B signaling pathway [27].

Taking this information into account, the effect of ouabain triggered by different inflammatory mediators related to carrageenan, zymosan, and compound 48/80-induced mouse paw inflammation was carried out. The present results demonstrate that ouabain produced a significant reduction in the mouse paw edema induced by prostaglandin E2 and bradykinin (at 15 minutes) but did not interfere in the edema induced by histamine. These findings suggest that the antiinflammatory effect of ouabain might be related to the inhibition of prostaglandin E2, bradykinin, and mast-cell degranulation but not to histamine. 

Another feature of acute inflammation is cell migration, primarily neutrophils, into the site of injury. The inhibitory effect of ouabain on neutrophil migration was confirmed through peritoneal inflammation induced by concanavalin-A. This mitogen possesses chemotactic activity for neutrophils [49] and ouabain produced a clear inhibition in the influx of polymorphonuclear cells to the peritoneal cavity. Together with the previous data, this result clearly demonstrates that ouabain has an antiinflammatory effect.

Prostaglandins are potent sensitizing agents able to modulate multiple sites of nociceptive pathways [50]. Prostaglandin E2 acts synergistically with bradykinin in the induction of pain. These substances are among the most important mediators of the inflammatory hyperalgesia [51–53]. Our results demonstrated that ouabain produced a significant reduction in the mouse paw edema induced by prostaglandin E2 and bradikynin. These data prompted us to evaluate the role of Ouabain in the nociception. 

The acetic acid-induced abdominal writhing is a sensitive method to evaluate peripherally acting analgesics. In this model, opioid mechanisms and released arachidonic acid via cyclooxygenase and prostaglandin biosynthesis play a role in the nociceptive mechanism [54–56]. In the present work, it was demonstrated that ouabain and morphine, caused a significant inhibition, 45% and 78%, respectively, on the writhing responses induced by acetic acid when compared to the control group. This peripheral analgesic potential might be related to the inhibition of PGE2 and bradykinin, as we have also found that ouabain reduced the edema formation induced by these mediators, suggesting that ouabain antinociceptive effect can be associated with an antiinflammatory action. 

To better evaluate the role of ouabain in pain, we used the thermal model for nociception, the hot-plate test, which is specific central antinociceptive test [57–59]. Ouabain was found to have antinociceptive activity in the hot-plate test, increasing the latency time (74%) when tested 30 min but not 60 nor 120 min after the last injection of ouabain. Animals were also pretreated with naloxone, an opioid antagonist that opposes the effects of opioid agonists such as morphine [37]. The results demonstrated that naloxone was capable to inhibit ouabain effect, indicating that at least in part, the antinociceptive effect of ouabain is also related to activation of opioid receptors. Using different models, it was also reported by other groups that ouabain exhibited antinociceptive effects [60, 61].

In an attempt to discard the possibility that the effect observed was due to a sedative effect on the animal behavior and not an analgesic effect, further experiments were performed, and mice were exposed to the elevated plus-maze test [38, 62–64]. This test is based on the natural fear of open and elevated spaces, and, as a consequence, rats and mice tend to avoid the open arms and stay on the enclosed arms [65, 66]. It was reported that an increase in the proportion of time spent in the open arms and in the proportion of entries into the open arms indicates anxiety reduction [38]. On the other hand, total number of arm entries is an indicator of motor activity [62–64]. Our data demonstrated that ouabain did not interfere in the mice anxiety behavior or in the motor function, when compared to the control group. As expected, the positive control (0.5 mg/Kg diazepan) leads to anxiety reduction demonstrated by an increase in the number of visits and time spent in the open arms and by an increase in the total number entries. These results suggest that the analgesic effect of ouabain is not related to sedative effect or to motor function.

## 5. Conclusion

For the first time, the present work demonstrated, *in vivo*, the antiinflammatory and analgesic potential of ouabain which might be related to prostaglandin E2 and bradykinin as well as to opioid mechanisms.
